# Abdominal multi-organ auto-segmentation using 3D-patch-based deep convolutional neural network

**DOI:** 10.1038/s41598-020-63285-0

**Published:** 2020-04-10

**Authors:** Hojin Kim, Jinhong Jung, Jieun Kim, Byungchul Cho, Jungwon Kwak, Jeong Yun Jang, Sang-wook Lee, June-Goo Lee, Sang Min Yoon

**Affiliations:** 1 Department of Radiation Oncology, Asan Medical Center, University of Ulsan College of Medicine, Seoul, 05505 Republic of Korea; 2 Department of Convergence Medicine, Asan Medical Center, University of Ulsan College of Medicine, Seoul, 05505 Republic of Korea

**Keywords:** Radiotherapy, Liver cancer

## Abstract

Segmentation of normal organs is a critical and time-consuming process in radiotherapy. Auto-segmentation of abdominal organs has been made possible by the advent of the convolutional neural network. We utilized the U-Net, a 3D-patch-based convolutional neural network, and added graph-cut algorithm-based post-processing. The inputs were 3D-patch-based CT images consisting of 64 × 64 × 64 voxels designed to produce 3D multi-label semantic images representing the liver, stomach, duodenum, and right/left kidneys. The datasets for training, validating, and testing consisted of 80, 20, and 20 CT simulation scans, respectively. For accuracy assessment, the predicted structures were compared with those produced from the atlas-based method and inter-observer segmentation using the Dice similarity coefficient, Hausdorff distance, and mean surface distance. The efficiency was quantified by measuring the time elapsed for segmentation with or without automation using the U-Net. The U-Net-based auto-segmentation outperformed the atlas-based auto-segmentation in all abdominal structures, and showed comparable results to the inter-observer segmentations especially for liver and kidney. The average segmentation time without automation was 22.6 minutes, which was reduced to 7.1 minutes with automation using the U-Net. Our proposed auto-segmentation framework using the 3D-patch-based U-Net for abdominal multi-organs demonstrated potential clinical usefulness in terms of accuracy and time-efficiency.

## Introduction

Radiotherapy for tumors in the upper abdomen is challenging because the tolerance radiation doses of the gastrointestinal tract and liver are not high enough to achieve the elimination of gross tumor burden. Recent improvements in radiotherapy and imaging have allowed the delivery of optimal radiation doses to upper abdominal tumors, especially hepatocellular carcinoma, while minimizing radiation dose to surrounding normal organs^1–3^. For more accurate radiotherapy and calculating dose distribution, delineation of the tumor and normal organs in computed tomography (CT) is necessary. However, tumor and organ delineation is notably laborious and has a steep learning curve^4^.

To increase the efficiency of organ segmentation, auto-segmentation methods as statistical shape models and atlas-based methods have been developed^5–10^. The statistical shape model is a knowledge-based method in which the atlases with majority voting depending on the image similarity are analyzed for capturing the anatomical information. Unfortunately, the atlas-based methods are likely affected by image deformation, and may overlook patient variability on normal anatomical structures. Therefore, these models have limited clinical applications.

The convolutional neural network is a recent breakthrough in deep learning technology that significantly improved the performance of normal organ auto-segmentation^11–13^. The U-Net with skip connections is a more developed type of convolutional neural network that was developed according to the variations in network architecture^14–17^. Although the U-Net has shown promising results for auto-segmentation in many studies^18–27^, additional enhancement and verifications are needed to make the U-Net comparable to radiographers in terms of segmentation accuracy. Moreover, only few studies on abdominal multi-organ segmentation have shown clinically applicable results in the abdomen, where normal organs are surrounded by soft tissue and vary in shape and location. The use of the U-Net to auto-segmentation of abdominal organs significantly improved its clinical applicability^24–27^. Therefore, we constructed a framework for abdominal multi-organ segmentation with the U-Net and compared its clinical usefulness by comparing it with the atlas-based segmentation and manual contouring by experienced radiographers.

## Results

### Clinical characteristics

Structure sets of 120 patients with hepatocellular carcinoma were used for training, validation, and testing. The median age of the patients was 59 years (range, 37–83) and males were dominant (81.7%) (Table 1). Ninety-three patients (77.5%) had liver functions of Child-Pugh class A, and 13.3% had mild-to-moderate degrees of ascites. Macroscopic vascular invasion was observed in 88 (73.3%) patients. Eight (6.7%) patients were treatment-naïve, and other patients had received various courses of treatments prior to radiotherapy; as a result, various changes such as iodized oils, low-density cavity after ablative therapy, and volume loss after hepatic resection existed in the livers of most patients.

**Table 1:** Patient characteristics.

Characteristics		No. of patients (n = 120)
Age (years)	Median (range)	59 (37–83)
Sex	Male	98 (81.7%)
	Female	22 (18.3%)
Child-Pugh classification	A	93 (77.5%)
	B	27 (22.5%)
Ascites	No	104 (86.7%)
	Yes	16 (13.3%)
Stage	Early	32 (26.7%)
	Advanced	88 (73.3%)
Vascular invasion	No	32 (26.7%)
	Yes	88 (73.3%)
Previous treatments	No	8 (6.7%)
	Yes	112 (93.3%)
	Total number, range	0–19
	Surgery	0–2
	RFA	0–5
	PEI	0–1
	TACE	0–16
	Radiotherapy	0–2

RFA, radiofrequency ablation; PEI, percutaneous ethanol injection; TACE, transarterial chemoembolization.

### Dice similarity coefficients and hausdorff distances

**Figure 1:**
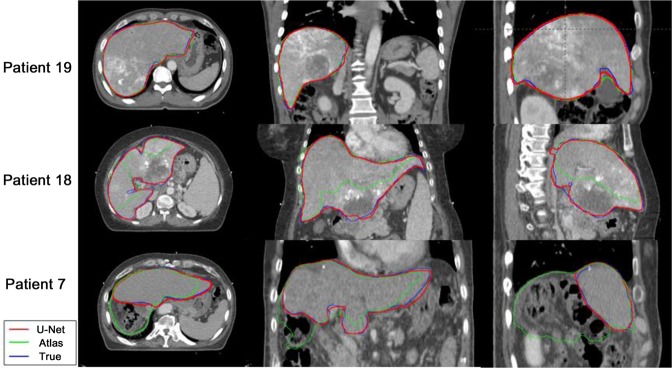
Comparison of liver contours in three testing cases. The U-Net-based segmentation (red), the atlas-based segmentation (green), and ground-truth manual contouring (blue) are shown.

Figure 1 shows the comparisons in the liver contours generated by the U-Net-based (red line) and the atlas-based (green line) segmentations to the ground-truth contour (blue line) in the three testing cases. Both auto-segmenting methods showed good agreement with the ground-truth contour in one case (patient 19), but the U-Net-based segmentation was notably superior in the other two cases, who had a low attenuated lesion of hypo-vascular infiltrative hepatocellular carcinoma in the liver (patient 18) and who underwent resection before radiotherapy (patient 7).

Table 2 shows the Dice similarity coefficients (DSCs), Hausdorff distances (HDs), and mean surface distances (MSDs) of the five structures produced from the U-Net-based, atlas-based, and inter-observer segmentations, in which the *P*-values were calculated for comparisons between the U-Net-based segmentation and the atlas-based and inter-observer segmentations. Compared with the atlas-based segmentation, the U-Net-based segmentation had a significantly greater DSCs, HDs, and MSDs for the liver, stomach, duodenum, and kidneys (right and left) (all *P* < 0.05). In terms of DSCs and HDs, the segmenting accuracy of the U-Net-based segmentation was similar to that of the inter-observer segmentation in the liver and both kidneys. Of note, the difference between auto-segmentation of the stomach and the duodenum was statically significant in DSCs and not in HDs. In contrast, when comparing the MSDs, the auto-segmentation by U-Net behaved differently from the inter-observer even in the liver, and right kidney besides the stomach, while behaving similarly for the duodenum (*P* > 0.05).

**Table 2:** Mean and standard deviation (in parenthesis) of Dice similarity coefficients and Hausdorff distances for the five structures*.

			Liver	Stomach	Duodenum	Kidney (Rt)	Kidney (Lt)
Dice similarity coefficient	U-Net-based		0.959 (0.018)	0.813 (0.137)	0.595 (0.186)	0.900 (0.174)	0.911 (0.159)
	Atlas-based		0.808 (0.116)	0.431 (0.188)	0.153 (0.113)	0.742 (0.027)	0.700 (0.219)
	Inter-observer		0.963 (0.006)	0.903 (0.048)	0.732 (0.052)	0.938 (0.184)	0.912 (0.105)
	*P*-value	U-Net vs. Atlas	0.000	0.000	0.000	0.000	0.000
		U-Net vs. Inter-observer	0.330	0.031	0.006	0.622	0.694
Hausdorff distance	U-Net-based		8.926 (6.298)	19.890 (19.286)	23.423 (20.088)	4.780 (2.735)	5.789 (4.278)
	Atlas-based		34.932 (18.619)	41.498 (23.814)	44.165 (14.096)	16.554 (8.995)	18.093 (12.228)
	Inter-observer		7.261 (2.476)	12.106 (9.363)	30.264 (12.692)	4.201 (1.299)	6.426 (7.522)
	*P*-value	U-Net vs. Atlas	0.000	0.007	0.003	0.000	0.000
		U-Net vs. Inter-observer	0.430	0.090	0.083	0.694	0.738
Mean surface distance	U-Net-based		0.714 (0.301)	3.034 (5.687)	2.796 (1.904)	1.104 (1.108)	0.918 (1.276)
	Atlas-based		4.114 (2.929)	8.115 (4.739)	13.661 (8.583)	2.989 (2.368)	3.680 (3.791)
	Inter-observer		0.460 (0.093)	0.743 (0.424)	2.944 (1.725)	0.665 (0.438)	0.781 (1.193)
	*P*-value	U-Net vs. Atlas	0.000	0.000	0.000	0.000	0.000
		U-Net vs. Inter-observer	0.000	0.001	0.679	0.030	0.143

*Produced from U-Net based method, Atlas-based method, and inter-observer, relative to the previously drawn ground-truth contours.

**Figure 2:**
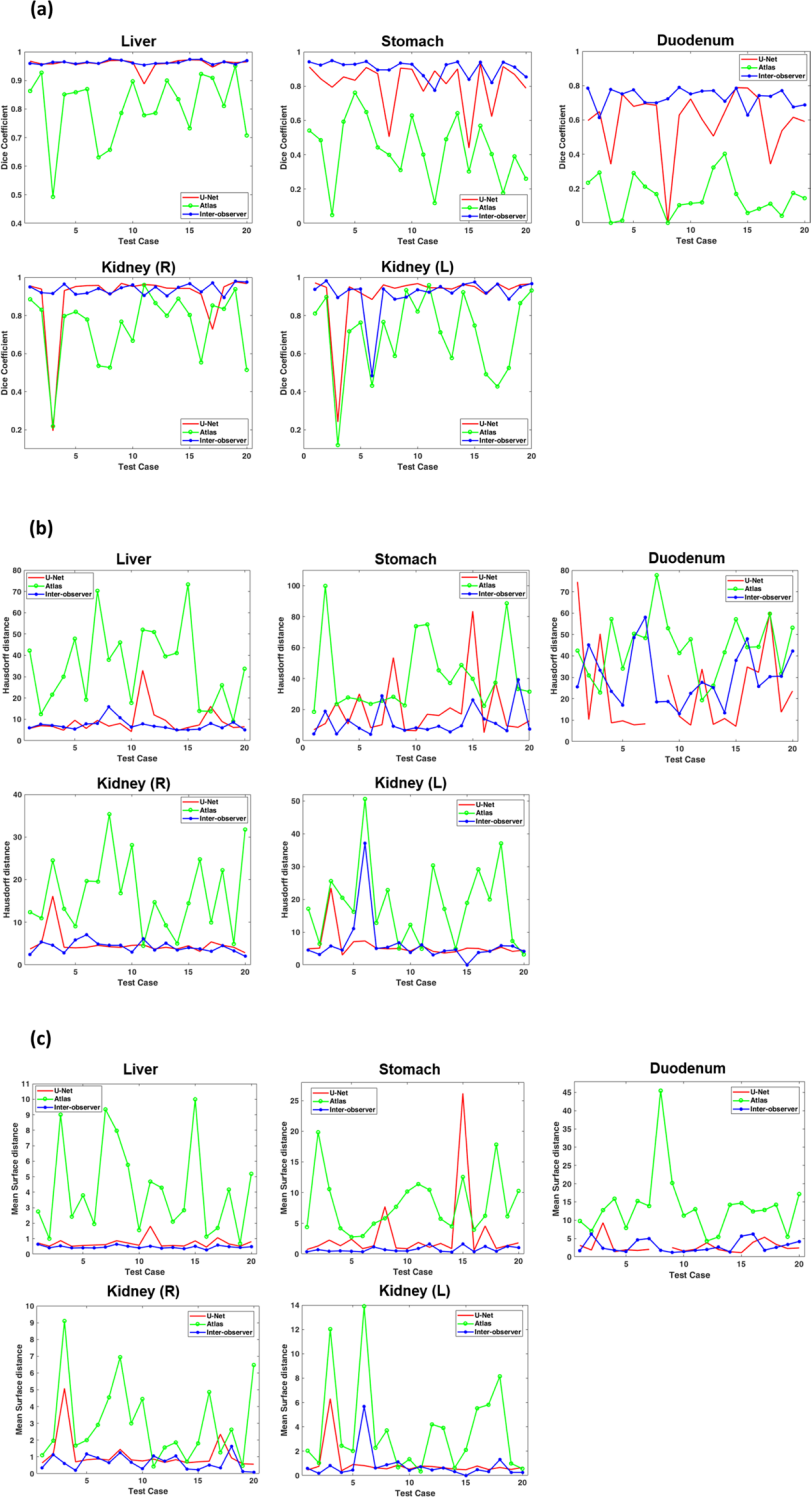
(**a**) Dice similarity coefficient (**b**) Hausdorff distance and (**c**) mean surface distance of the five structures (liver, stomach, duodenum, and right/left kidneys) produced by the U-Net-based segmentation, the atlas-based segmentation, and the inter-observer segmentation, relative to the previously drawn ground-truth manual contours in 20 testing cases.

Figure 2 shows the DSCs (Fig. 2a), HDs (Fig. 2b), and MSDs (Fig. 2c) of the U-based, the atlas-based, and inter-observer segmentations for five structures of the 20 testing cases. From the resulting HDs, MSDs, and DSCs of the inter-observer delineating contours against the ground-truth, inter-observer variability was observed in the metrics, which was especially notable in the duodenum. For the liver and kidneys, the U-Net-based segmentations had similar DSCs, HDs, and MSDs to the inter-observer segmentations; specifically, in the liver, the DSC and HD values of the U-Net-based segmentation were similar among different patients, except for patient 11 in whom the liver had extended to the spleen region due to hypertrophy of the left lateral segment. Patient 3 had a notably low accuracy of kidney segmentations due to the relatively insufficient enhancement on CT images. Except for this case, the DSC reached 0.937 and 0.947 for right and left kidneys, respectively, and the U-Net did not show a statistically significant difference for the MSDs of the right kidney (*P* = 0.055). Although the U-Net-based segmentation outperformed the atlas-based segmentation, it did not reach the expert level (inter-observer delineation) in the stomach and duodenum. Overall, the DSC values more clearly and stably showed the relative superiority of each segmentation method, while the HD and MSD values were relatively noisy and fluctuating.

### Efficiency in real-life clinical practice

**Figure 3:**
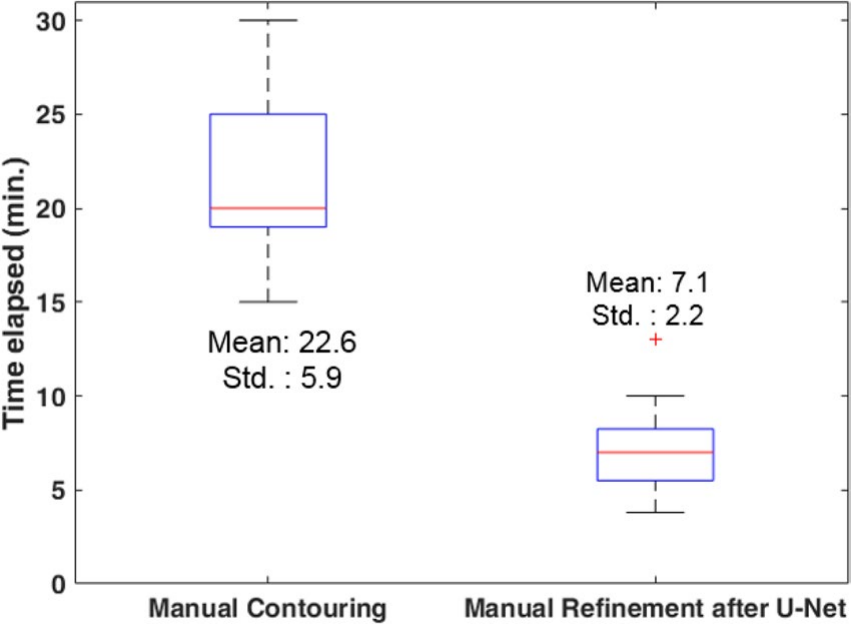
Comparisons of the time elapsed for manual contouring and manual refinement after the U-Net segmentation.

To investigate the efficiency of the U-Net-based segmentation, we compared the elapsed segmentation times for manual contouring without automation (*n* = 20) with the time for refinement of the structures generated from the U-Net (*n* = 20). The segmentation time was significantly lower in manual refinement after the U-Net-based segmentation (Fig. 3): the mean (standard deviation, median, interquartile range) elapsed times in manual contouring and manual refinement after the U-Net segmentation were 22.6 (5.9, 20.0, 19.0–25.0) and 7.1 (2.2, 7.0, 5.5–8.3) minutes, respectively (*P* < 0.001). The average structure-specific additional times for refinement were 2.0, 2.0, 2.5, and 1.0 minutes for liver, stomach, duodenum, and right/left kidneys, respectively. Notably, segmentation refinement of the duodenum required the longest time due to the relatively inferior accuracy of auto-segmentation. In contrast, owing to the superior performance of liver auto-segmentation, the additional manual refinement time needed was approximately 2 minutes for the liver, the largest organ in the upper abdomen.

## Discussion

Auto-segmentation for normal organs has been a demanding process in radiotherapy, and the advent of the deep convolutional neural network involving 2D or 3D images in training big data brought forth significant improvement in auto-segmentation. We utilized the 3D-patch-based U-Net framework combined with graph-cut post-processing for multi-organ segmentation in the abdomen; as a result, our proposed automated framework based on the U-Net outperformed the previously developed atlas-based auto-segmentation for all contours in terms of DSCs, HDs, and MSDs. Although not showing the same statistical trend as the MSDs, the U-Net-based segmentation also showed comparable accuracy to the ground-truth segmentations in the liver and kidneys with relatively high image intensity gradients in terms of DSCs and HDs.

We noticed some outliers in the study, which mostly occurred due to extensive liver volume expansion and insufficient kidney enhancement on CT images, which could be complemented by increasing the number of such cases. The accuracy of the U-Net-based segmentation was lower in the stomach and duodenum, which was expected as they had larger inter-observer variability as well. As such, the DSC values of the U-Net-based segmentation were significantly lower than those of the inter-observer segmentations. Interestingly, the mean of maximum surface distance in the duodenum was smaller in the U-Net-based segmentation than in the inter-observer segmentation.

The quality of the proposed U-Net-based segmentation framework is difficult to quantify in relation to the previous attempts throughout the deep neural network. The DSCs of liver contouring ranged from 0.94 to 0.98 in recent publications^24–33^, and the inter-observer variability in liver DSC was reported as 0.96^9^, which is very close to the 0.959 in our study. Considering that most patients in our study had advanced-stage hepatocellular carcinomas with a history of multiple treatment sessions, the liver DSC of our auto-segmentation framework at 0.959 can be regarded as a promising result. Also, the U-Net-based segmentation significantly reduced the time required for five abdominal organ segmentation, as manual refinement after U-Net-based segmentation only took approximately one-third of the time required in manual contouring. Such time benefit was the greatest for segmentation of the liver, the largest visceral organ in the abdomen, which only required 2 minutes on average for additional refinement. Though not included in the manuscript, we measured the refinement time after atlas-based segmentation and found that the atlas-based segmentation required a significantly longer refinement time than the manual contouring and was thus deemed inapplicable. Collectively, the U-Net-based segmentation seems sufficiently accurate and highly efficient in segmenting abdominal organs and thus may be regarded to have adequate clinical applicability. At our clinic, the U-Net-based segmentation framework is being routinely used for auto-segmentation of normal organs in patients with liver cancer; moreover, the manually refined contours are stored in our intranet server so that they may be updated and improved by additional training to meet the demands of each user.

The study has several areas of limitation. First, our 3D U-Net based segmentation followed by the graph-cut post-processing showed a relatively weak performance in the stomach or duodenum, which have low intensity gradients. Several recent studies reported better results by overcoming these pitfalls through multi-resolution approach^28,29^, multi-planar statistical fusion^30,31^, and semi-supervised training with both labeled and unlabeled output data^32,33^. Thus, by utilizing iterative or additional training stages, more promising results may be achieved for the stomach and duodenum. Hence, the current form could be evolved and achieve additional time efficiency. Second, this study focused on normal organs only in the upper abdomen and not the whole body. Nevertheless, normal organs in the upper abdomen are considered the most challenging in auto-segmentation technique, especially due to the lack of inherent contrast differences between the organs and poor image quality due to the respiratory movement during CT scans. Therefore, the promising result of the present study indicates that auto-segmentation in the whole body may be possible and should be investigated in future studies. Third, we set the segmentation results from the radiographers as the gold standard. As manual contouring may be inherently limited due to human conditions as well as differences in contour devices, the true performance of the U-Net-based segmentation for normal organs should be further evaluated. Fourth, due to the extensive amount of experience of our radiographers, the mean time elapsed for manual contouring of five abdominal structures was quite short at 23 minutes, which may not be readily achievable in smaller-sized institutions. Therefore, the degree of additive efficiency of assistance by auto-segmentation may be more prominent in other institutions. It may be reasonable to assume that the final check and refinement process would take less than 10 minutes for five abdominal structures; moreover, this time may be further reduced by additional training with the manually refined contours as mentioned above. Lastly, only the time reduction parameter was used to show the efficiency of auto-segmentation. Future studies should assess other aspects of the usefulness of the auto-segmentation system such as differences in the degree of physical comfort or streamlining of the work process.

In conclusion, the auto-segmentation framework consisting of 3D-patch-based U-Net and graph-cut algorithm was superior to the atlas-based segmentation and comparable to manual contouring in terms of accuracy in segmenting abdominal organs. Moreover, the U-Net-based segmentation significantly augmented the clinical efficiency of manual contouring by reducing the time required for segmentation. Our proposed workflow would be particularly useful in patients with liver cancer for auto-segmenting their abdominal organs, the most challenging organs for auto-segmentation.

## Methods

### Patient selection

Anatomic structures of patients who were treated with radiotherapy for hepatocellular carcinoma between July 2017 and March 2018 at Asan Medical Center (Seoul, Korea) were collected in order to train for various complicated normal organ conditions containing primary tumors in the liver. This study was approved by the Institutional Review Board of Asan Medical Center, and the need for informed consent was waived considering the retrospective nature of the study. This study was conducted in accordance with the Declaration of Helsinki.

### CT simulation and multi-organ segmentation

The simulation and segmentation procedures had been described in our previous report^3^. All patients were immobilized in the supine position using a vacuum cushion and underwent a free-breathing 4-dimensional (4D) CT scan (GE LightSpeed RT 16; GE Healthcare, Waukesha, WI, USA). A real-time position management respiratory gating system (Varian Medical Systems, Palo Alto, CA, USA) was used to record the patients’ breathing patterns. The CT slice thickness was set to 2.5 mm. An intravenous contrast agent was injected to improve the segmentation accuracy of the target and normal organs. The CT data were sorted into 10 CT series according to the respiratory phase using 4D imaging software (Advantage 4D version 4.2; GE Healthcare). Contouring was performed on the CT images at the end-expiratory phase using a radiotherapy treatment planning system (Eclipse version 13.6; Varian Medical Systems). The organs at risk included the whole liver, duodenum, stomach, and right/left kidneys. Two professional radiographers with experience in segmentation of abdominal structures of more than 500 patients per year delineated the training sets and two radiation oncologists with more than 10 years of experience in hepatocellular carcinoma treatment confirmed each structure.

### Deep neural network and post-processing

**Figure 4:**
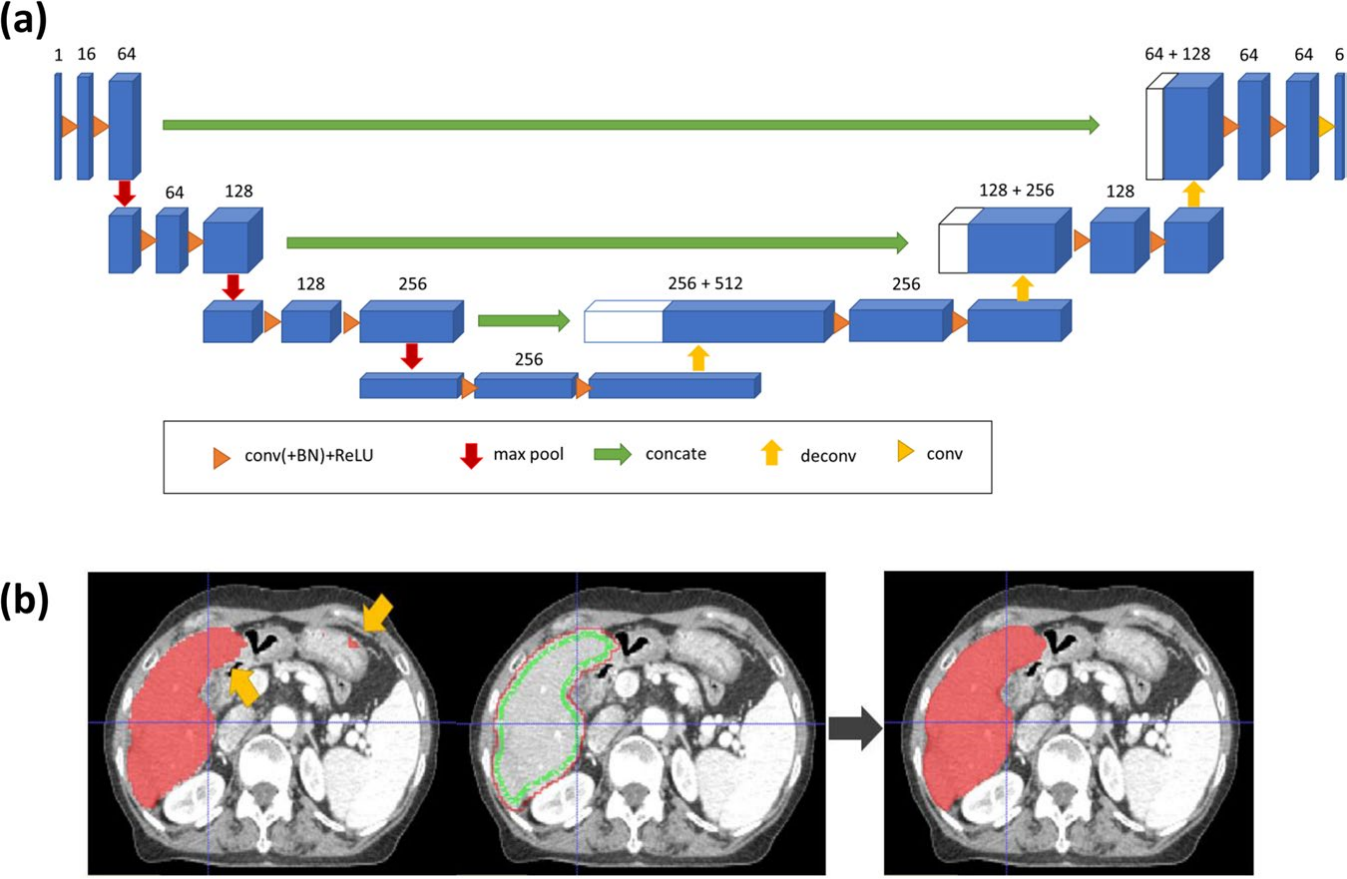
Proposed auto-segmenting framework. Network architecture of the (**a**) 3D-patch-based U-Net and (**b**) Graph-cut post-processing. Liver segmentation is shown as an example.

The basic network architecture for multi-organ auto-segmentation in the abdomen was based on the 3D U-Net^16,17^ as illustrated in Fig. 4a. The network was designed to input the 3D-patch extracted from volumetric CT images, and output the 3D-patch from multi-label semantic images. It interpolated the 3D CT images to have 2 mm resolutions for x, y, and z directions. The network input consisted of 3D-patch-based CT images with 64 × 64 × 64 voxels, which were randomly sampled for training procedure. The loss function of the network was defined as the cross-entropy loss function, and the optimizer was set to be RMSprop. The training was performed with 8 mini-batches of 3D-patch at 10^−5^ learning rate and 10^5^ iterations.

To improve the segmenting accuracy, we added post-processing based on a graph-cut algorithm^34,35^, which is composed of data penalty for regional label preferences and spatial coherence for boundary by penalizing intensity discontinuity (gradient). For the U-Net-generated liver auto-segmented structures, the false-positive regions were removed by the data penalty term and the organ boundary was delineated by the boundary term from discontinuity penalization (Fig. 4b).

### Training, validating, and testing of the network

The proposed framework of the U-Net and post-processing was trained with 80 cases and validated with 20 cases, without cross-validation. We assessed the performance of the proposed auto-segmenting framework (3D-patch-based U-Net and graph-cut refinement) for 20 test datasets that were not included in the training and validation set. The 20 test datasets manually drawn by the radiographers were regarded as the reference ground-truth segmentations in the performance check for the above-mentioned five structures. The proposed auto-segmenting framework was compared against the auto-segmented structures predicted by the atlas-based method (Mirada Medical, Oxford, UK), of which the atlas-liver library consisted of 20 CT simulation scans. Also, to assess the inter-observer variability between radiographers and to estimate the efficiency of auto-segmentation by U-Net, the contours of the 5 selected structures of the 20 test cases were manually delineated by a radiation oncology resident who did not conduct segmentation on the same datasets.

The training of the convolutional neural network was implemented using parallel computing architecture on an Intel Core i9-7960 CPU (2.8 GHz) and a single GPU-enabled Nvidia GeForce RTX Titan graphics card (24 GB memory). The training of the 3D patch-based U-Net model took 3–4 days in 10^5^ iterations with the TensorFlow (version 1.14) library. The pre-processing (i.e., interpolation and intensity normalization) and post-processing (i.e., graph-cut algorithm) steps were implemented using the Insight Segmentation and Registration Toolkit library.

### Evaluation of the segmenting accuracy and clinical applicability

The accuracies of the two auto-segmentation methods (U-Net-based and atlas-based) and the manual inter-observer segmentation were quantified by three indices—DSC, HD, and MSD. The DSC is the volumetric comparison between the two structures, the HD is the maximum of the minimum of surface distances between the two resulting contours, and the MSD is the average of the minimum surface distances between two resulting contours. The DSCs describe how similarly the predicted contours are shaped, while the HDs and MSDs indicate how much the contours should be quantitatively modified relative to the ground-truth. Statistical analysis was also performed to determine if the results from our proposed U-Net-based auto-segmenting framework can generate statistically significant differences relative to the atlas-based and inter-observer segmentations.

Besides accuracy, the clinical applicability in terms of time efficiency was also analyzed. It was assumed that additional manual revision on the U-Net based auto-segmentation is needed to reach the level of human experts. Thus, we measured the time elapsed for additional refinement for the predicted contours patient-by-patient and organ-by-organ basis in the 20 independent cases. A professional radiographer manually revised the five structures predicted by the U-Net-based segmentation. The extra time was compared against the manual contouring time with no aid to the auto-segmentation platform.
